# Decreased LKB1 predicts poor prognosis in Pancreatic Ductal Adenocarcinoma

**DOI:** 10.1038/srep10575

**Published:** 2015-05-27

**Authors:** Jian-Yu Yang, Shu-Heng Jiang, De-Jun Liu, Xiao-Mei Yang, Yan-Miao Huo, Jiao Li, Rong Hua, Zhi-Gang Zhang, Yong-Wei Sun

**Affiliations:** 1 Department of Biliary-Pancreatic Surgery, Ren Ji Hospital, School of Medicine, Shanghai Jiao Tong University, 200240 Shanghai, P.R. China; 2 State Key Laboratory of Oncogenes and Related Genes, Shanghai Cancer Institute, Ren Ji Hospital, School of Medicine, Shanghai Jiao Tong University, 200240 Shanghai, P.R. China; 3 Shanghai Medical College of Fudan University, Shanghai 200032, P.R. China

## Abstract

Liver kinase B1 (LKB1) has been identified as a critical modulator involved in cell proliferation and polarity. The purpose of the current study was to characterize the expression pattern of LKB1 and assess the clinical significance of LKB1 expression in pancreatic ductal adenocarcinoma (PDAC) patients. LKB1 mRNA expression which was analyzed in 32 PDAC lesions and matched non-tumor tissues, was downregulated in 50% (16/32) of PDAC lesions. Similar results were also obtained by analyzing three independent datasets from Oncomine. Protein expression of LKB1 was significantly reduced in 6 PDAC cell lines and downregulated in 31.3% (10/32) of PDAC lesions compared to matched non-tumorous tissues, as determined by Western blot analysis. Additionally, tissue microarray containing 205 PDAC specimens was evaluated for LKB1 expression by IHC and demonstrated that reduced expression of LKB1 in 17.6% (36/205) of PDAC tissues was significantly correlated with clinical stage, T classification, N classification, liver metastasis and vascular invasion. Importantly, Kaplan-Meier survival and Cox regression analyses were executed to evaluate the prognosis of PDAC and found that LKB1 protein expression was one of the independent prognostic factors for overall survival of PDAC patients.

Pancreatic ductal adenocarcinoma (PDAC) remains one of the most fatal malignancies, and patients diagnosed with PDAC have a mortality rate that closely equals morbidity^1^. Most patients are not eligible for surgical resection due to occult progression of this aggressive disease. The clinical course is extremely dire, with an overall 5-year survival rate of approximately 6%^2^. In order to improve the survival and quality of life of these patients, novel therapeutic approaches and prognostic factors must be researched and developed to improve clinical outcomes in patients with PDAC.

Liver kinase B1 (LKB1, STK11) is key regulatory protein of cellular metabolism that was originally identified in patients with Peutz-Jeghers syndrome (PJS), an autosomal dominant disease associated with LKB1 germline alterations^3^. Patients with PJS are predisposed to both benign and malignant cancers in multiple organ systems^4^,^5^,^6^,^7^ (including gastrointestinal, gynecologic, breast and pancreatic cancers). LKB1 acts via the AMP-activated protein kinase (AMPK) pathway, and is frequently lost in sporadic pancreatic cancer and lung adenocarcinoma^8^,^9^. Inactivation of LKB1 results in the activation of the mammalian target of rapamaycin (mTOR) pathway, which is crucial in controlling cellular energy metabolism, cell survival and growth under metabolic stress such as nutrient deficiency^10^. The PDAC tumor microenvironment is typically characterized by a diminished vascular network in the setting of striking desmoplastic response, and, under these conditions, LKB1 inactivation may be predicted to have a pro-tumorigenic response.

Previous studies of LKB1 in pancreatic acinar cell carcinoma^11^, intraductal papillary-mucinous neoplasms (IPMN)^12^,^13^ and PDAC^7^ have focused on germline or somatic mutations and inactivation. In addition to mutations, allelic loss and promoter hypermethylation also have been reported as mechanisms that account for LKB1 loss in a variety of tumors^14^. However, the effect of these genetic and epigenetic alterations on LKB1 expression in large numbers of sporadic PDAC remain unclear.

In this retrospective study, we examined the expression pattern of LKB1 at both the mRNA and protein level and explored the relationship of LKB1 expression with clinicopathologic parameters, including overall survival. We found that the expression of LKB1 was associated with poor prognosis of PDAC.

## Results

### LKB1 mRNA expression in PDAC

**Figure 1:**
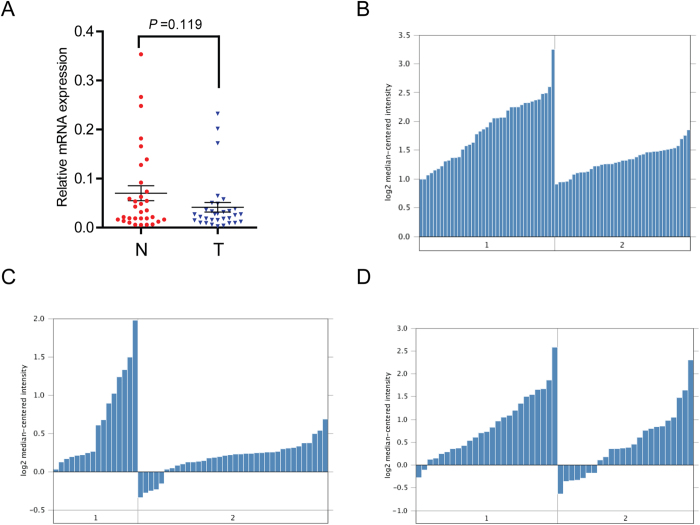
LKB1 expression is decreased in PDAC at mRNA level. A Decreased LKB1 mRNA expression in 32 matched tumor (T) and non-tumor tissue (N) was detected by Real-time quantitative PCR. LKB1 expression in Pei pancreas (**B**), Ishikawa pancreas (**C**) and Badea pancreas (**D**) grouped by normal pancreatic duct (1) and PDAC (2).

LKB1 mRNA expression levels were examined using real-time quantitative PCR in 32 PDAC tumor tissues and compared to levels from patient-matched adjacent non-tumor tissues. The results demonstrated that LKB1 mRNA expression was downregulated in 50% (16/32) of PDAC patients (Fig. 1A). Additionally, we analyzed three independent microarray datasets from Oncomine database^15^,^16^,^17^ for LKB1 expression (Fig. 1B–D). Within the datasets, mRNA expression levels of LKB1 were downregulated in the majority of tumor tissues compared with normal pancreatic duct was consistent with our data.

### LKB1 protein expression in PDAC

**Figure 2:**
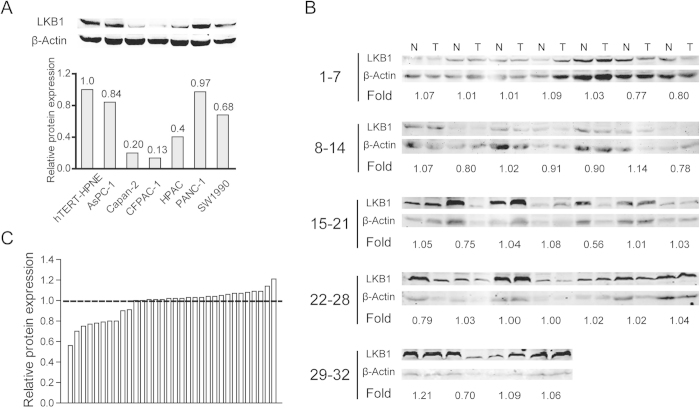
Western blotting analysis of LKB1 expression in PDAC cell lines and 32 pairs of resection specimens from PDAC patients. (**A**) Western blots show significantly reduced protein expression of LKB1 in 6 PDAC cell lines compared to the nonmalignant hTERT-HPNE cells. (**B**) Results of western blotting analysis of LKB1 protein expression in 32 pairs of resection specimens from PDAC patients. (**C**) The relative LKB1 protein expression levels in 32 pairs of representative PDAC tumor tissues and the matched adjacent non-tumor tissues.

**Figure 3:**
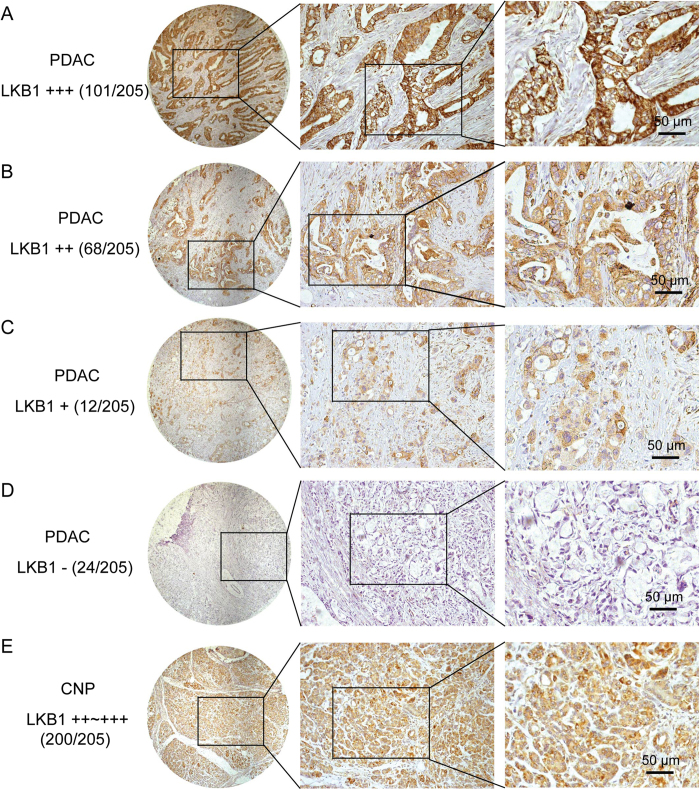
LKB1 expression in PDAC tissue samples. **A-E**, Representative images of LKB1 expression in PDAC compared with corresponding noncancerous pancreas (CNP); (**A**) PDAC, scored as (+++); (**B**) PDAC, scored as (++); (**C**) PDAC, scored as (+); (**D**) PDAC, scored as (-); (**E**) CNP, scored as (++~+++).

LKB1 protein levels were evaluated using Western blot or immunohistochemical staining (IHC). The expression of LKB1 protein was decreased in all the six PDAC cell lines compared with the nonmalignant hTERT-HPNE cells (Fig. 2A). However, in 32 pairs of resection specimens (tumor tissues and matched adjacent non-tumor tissues) from PDAC patients, a decrease in LKB1 expression was observed in 31.3% (10/32) of the PDAC tumor tissues compared with the matched adjacent non-tumor tissues (Fig. 2B,C). LKB1 protein expression was then evaluated by IHC using a tissue microarray containing 205 paired PDAC samples. This set of experiments showed that LKB1 protein was downregulated in 17.6% (36/205) of PDAC tissues and 2.4% (5/205) of corresponding normal tissues, respectively (Fig. 3). A statistically significant difference was observed between LKB1 expression in PDAC tumor tissues and their matched adjacent non-tumor tissues (*P* < 0.001).

### Relationship between LKB1 expression and clinical parameters

To evaluate the clinical significance of LKB1 expression in PDAC, the *Chi-square* test was used to assess the correlations between LKB1 protein expression and clinicopathologic parameters (including age, gender, size, tumor location, clinical stage, T classification, N classification, liver metastasis, vascular invasion and Histological differentiation). The results demonstrated that LKB1 expression in PDAC tissues is closely correlated with clinical stage (*P* = 0.001), T classification (*P* = 0.025), N classification (*P* = 0.015), liver metastasis (*P* = 0.005) and vascular invasion (*P* = 0.004). No significant associations were observed between LKB1 expression and age, gender, tumor size, tumor location or histological differentiation (Table 1).

### Correlation between LKB1 expression and prognosis in PDAC patients

**Figure 4:**
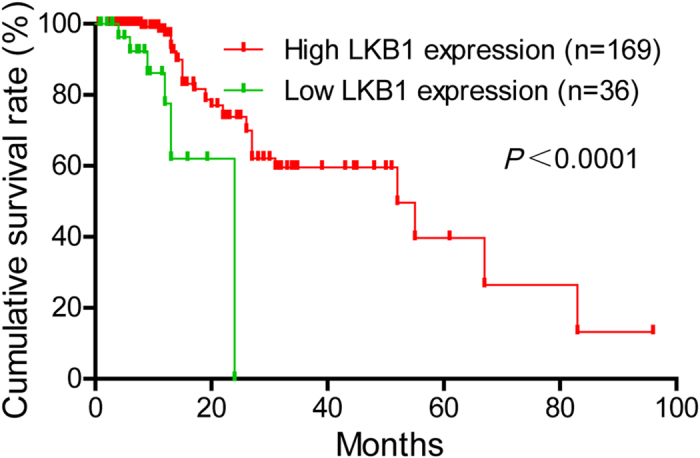
LKB1 expression is correlated with overall survival rate in PDAC patients. Kaplan-Meier survival curves show low expression level of LKB1 was significantly correlated with poor survival of PDAC. *P*-values were calculated by log-rank test.

**Figure 5:**
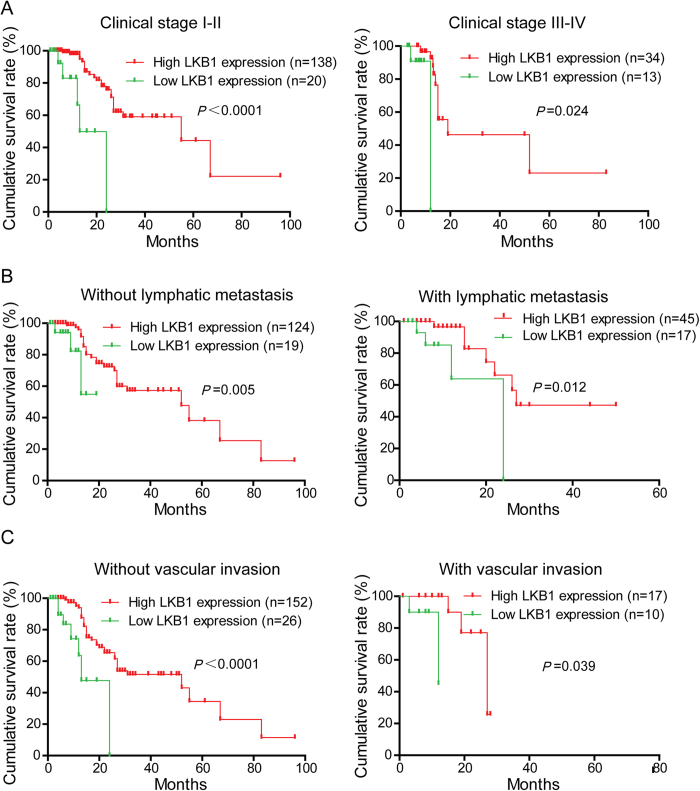
Correlation between LKB1 expression and overall survival rate in PDAC patients is independent of clinical stage, status of lymphatic metastasis and vascular invasion. A Comparisons of overall survival between in LKB1 low expression and LKB1 high expression in early clinical stage (I-II) cohort and in advanced clinical stage (III-IV) cohort. **B** Comparisons of overall survival between in LKB1 low expression and LKB1 high expression in patients with or without lymph node metastasis. C Comparisons of overall survival between in LKB1 low expression and LKB1 high expression in patients with or without vascular invasion. *P*-values were calculated by log-rank test.

To determine the prognostic value of LKB1 in PDAC, the relationship between LKB1 expression and clinical follow-up information was analyzed by Kaplan-Meier analysis and log-rank test. As shown in Fig. 4, low LKB1 expression was associated with poor overall survival (*P* < 0.001). In addition, we determined the correlation between LKB1 expression and overall survival in PDAC patients with early or advanced clinical stages and with or without lymphatic metastasis and vascular invasion. Kaplan-Meier analyses showed that overall survival is shorter in PDAC patients with lower LKB1 expression independent of clinical stage. Similar results were also found in PDAC patients with or without lymphatic metastasis or vascular invasion (Fig. 5). Univariate and multivariate analyses were performed to confirm the possibility that LKB1 could be useful as an independent risk factor for poor prognosis in the 205 cases of PDAC. Univariate Cox regression analyses showed that LKB1 expression, clinical stage, tumor size, T classification, N classification and Liver metastasis were significantly associated with overall survival (Table 2). Furthermore, a multivariate Cox regression analysis confirmed LKB1 expression, tumor size and Liver metastasis as independent predictors of the overall survival in patients with PDAC (Table 2).

## Discussion

Studies focused on human clinical specimens and genetically engineered mouse models of PDAC have lead to a better understanding of this genetic malignancy^18^,^19^. LKB1 has emerged as a major tumor suppressor in various tumor types, and it is well-described in the literature that LKB1 loss confers poor clinical outcome in many different cancers^14^,^20^. However, the literature lacks a comprehensive investigation remains to evaluate the clinical significance in PDAC specifically. Here, LKB1 expression and its correlation with clinicopathological features and clinical prognosis are reported.

Initially, LKB1 expression was assessed at mRNA and protein level. The data and three Oncomine databases collectively confirmed that LKB1 expression was reduced in PDAC tissues compared with adjacent non-tumor tissues or normal pancreatic duct cells at the mRNA level. Together, these observations indicated that LKB1 may function as tumor suppressor in PDAC. Furthermore, LKB1 protein level as analyzed by IHC and Western blot showed that LKB1 expression was reduced in 17.6% (36/205) and 31.3% (10/32) of PDAC tissues respectively. This finding is consistent with previous studies which demonstrated that LKB1 expression was decreased in around 20% of human PDAC^21^. Interestingly, our data demonstrated that LKB1 expression at protein level was much lower than may be predicted by mRNA levels alone, which was decreased in 50% (16/32) of PDAC patients. This may be accounted for the post-transcriptional modifications in LKB1 expression^22^. Additionally, there was a remarkably higher ratio of low LKB1 expression in PDAC cell lines (Fig. 2A) assessed by Western blot compared with immunohistochemical staining. The non-malignant hTERT-HPNE cells used in our study are intermediary cells formed during acinar-to-ductal metaplasia but not the normal pancreatic duct cell, and discrepancies of LKB1 expression in cell lines measured by Western blot are more sensitive than immunohistochemical staining in quantity may explain this difference.

The relationship between LKB1 expression levels and certain clinicopathologic parameters was evaluated. Previous research has shown that, in lung adenocarcinoma, LKB1 loss at the transcriptional level promotes tumor malignancy and consequently resulting in poor patient outcomes^20^. Our study revealed a similar phenomenon in which reduced LKB1 expression in PDAC was correlated with clinical stage, T classification, N classification, liver metastasis and vascular invasion. Importantly, patients with a low level of LKB1 expression had significantly shorter survival times compared to those with a high level of LKB1 expression.

Univariate analysis showed that reduced LKB1 expression was significantly associated with the overall survival rate in PDAC patients. Multivariate analysis indicated that LKB1 expression was an independent risk factor for poor prognosis of PDAC patients. Collectively, these results suggest that LKB1 might be used as a novel prognostic marker for PDAC patients. Of note, LKB1 detection assays in mouse and human tissues has been used in multiple investigations, revealing that LKB1 could be a potential predictor of clinical prognosis in diverse human malignancies^23^.

In conclusion, this study demonstrated that reduced LKB1 expression is associated with poor survival in PDAC patients, suggesting that LKB1 expression may serve as an important prognostic marker and may represent a potential molecular target for the treatment of PDAC.

## Materials and Methods

### Cell culture

Human PDAC cell lines AsPC-1, Capan-2, CFPAC-1, HPAC, PANC-1 and SW1990 were all preserved in Shanghai Cancer Institute and a normal control cell line, hTERT-HPNE, was purchased from American Type Culture Collection. All of these cells were cultured in specific medium supplemented with 10% (v/v) fetal bovine serum (FBS) and 1% antibiotics at 37°C in a humidified incubator under 5% CO_2_ condition.

### Clinical tissue samples and immunochemistry

Human PDAC tissue microarrays containing 205 cases of tumor and matched non-tumor tissues were all recruited between January 2002 and June 2014 from Ren Ji Hospital, School of Medicine, Shanghai Jiao Tong University, China. Clinicopathologic characteristics of patients are provided in Table 1. The histology and clinical stages were classified according the seventh edition of the American Joint Committee on Cancer (AJCC) staging system. The cases of PDAC were selected in this study only if clinical data were available. The follow-up time was calculated from the date of surgery to the date of death, or the last known follow-up. None of them had received radiotherapy, chemotherapy, hormone therapy or other related anti-tumor therapies before surgery. An additional 32 paired freshly frozen PDAC tissues and corresponding non-cancerous tissues were also obtained from Ren Ji Hospital, School of Medicine, Shanghai Jiao Tong University, China. All patients provided written informed consent, and the experiments were approved by the Hospital Research Ethics Committees of Ren Ji, School of Medicine, Shanghai Jiao tong University. All methods were carried out in accordance with the approved guidelines of School of Medical graduate Shanghai Jiao tong University. Immunohistochemical staining was performed as previous described^24^. Scoring of LKB1 expression was conducted according to the ratio of positive cells: 0-5% scored 0; 6%-35% scored 1; 36%-70% scored 2; more than 70% scored 3 and staining intensity: no staining scored 0, weakly staining scored 1, moderately staining scored 2 and strongly staining scored 3, respectively. The final score was designated as low or high expression group using the percent of positive cell score × staining intensity score as follows: “-” for a score of 0-1, “+” for a score of 2-3, “++” for a score of 4-6 and “+++” for a score of >6; low expression was defined as a total score <4 and high expression with a total score ≥4. These scores were determined independently by two senior pathologists. The scoring was done in a blinded manner by the pathologists.

### Real-time quantitative PCR

Total RNA from primary tumor and adjacent non-tumor tissue samples was extracted using Trizol reagent (Takara, Japan), and reversely transcribed using a PrimeScript RT-PCR kit (Takara, Japan) according to the manufacturer’s instructions. Quantitative real-time PCR was performed using a 7500 Real-time PCR system (Applied Biosystems, Inc. USA). Primer sequences sed for LKB1 detection were as follows, forward: 5′-AGGGATGCTTGAGTACGAACC-3′; reverse: 5′- GTCCTCCAAGTACGGCACC-3′. The relative expression of was normalized to â-actin RNA (forward: 5′-ACTCGTCATACTCCTGCT-3′, reverse: 5′- GAAACTACCTTCAACTCC-3′). The 2^−∆Ct^ method was used to quantify the relative LKB1 expression levels and normalized using the β-actin expression.

### Western blotting

Western blot analyses were performed as previously described^25^. LKB1 antibody was purchased from Proteintech Inc. and species-specific secondary antibody was purchased from Cell Signaling, Beverly, MA. Secondary antibodies were detected by Odyssey imaging system (LI-COR Biosciences, Lincoln, NE).

### Statistical analysis

Statistical analyses and graphical representations were performed using SPSS 16.0 (SPSS Inc.; Chicago, IL, USA) and GraphPad Prism 5 (San Diego, CA) software. The χ^*2*^ test was used to analyze the correlations between LKB1 expression and clinicopathologic features in patients with PDAC. Survival curves were evaluated using the Kaplan-Meier method, and differences between survival curves were tested by the log-rank test. Cox proportional hazards regression model was used to examine univariate and multivariate hazard ratios for the study variables that were dichotomized. Only significantly different variables in univariate analysis including LKB1 expression level, Clinical stage, Size, T classification, N classification, Liver metastasis were entered into the next multivariate analysis. A two-sided *P*-value < 0.05 was considered statistically significant.

## Additional Information

**How to cite this article**: Yang, J.-Y. *et al.* Decreased LKB1 predicts poor prognosis in Pancreatic Ductal Adenocarcinoma. *Sci. Rep.* **5**, 10575; doi: 10.1038/srep10575 (2015).

## Figures and Tables

**Table 1:** Correlations between LKB1 expression and clinicopathologic features in patients with pancreatic ductal adenocarcinoma (PDAC).

		**Expression of LKB1**		
**Clinicopathological feature**	**Total 205**	**Low (n = 36, 17.6%)**	**High (n = 169, 82.4%)**	**P value (χ^2^ test)**
**Age (years)**				
≤65	107	17 (15.9)	90 (84.1)	0.511
>65	98	19 (19.4)	79 (80.6)	
**Gender**				
Male	117	16 (13.7)	101 (86.3)	0.092
Female	88	20 (22.7)	68 (77.3)	
**Clinical stage (AJCC)**				
I	23	1 (4.3)	22 (95.7)	**0.001**
II	135	19 (14.1)	116 (85.9)	
III	34	14 (41.2)	20 (58.8)	
IV	13	2 (15.4)	11 (84.6)	
**Size**				
≤2 cm	41	3 (7.3)	38 (92.7)	0.054
>2 cm	164	33 (20.1)	131 (79.9)	
**T classification**				
T1	7	0 (0.0)	7 (100.0)	**0.025**
T2	31	1 (3.2)	30 (96.8)	
T3	129	24 (18.6)	105 (81.4)	
T4	38	11 (28.9)	27 (71.1)	
**N classification**				
Absent	143	19 (13.3)	124 (86.7)	**0.015**
Present	62	17 (27.4)	45 (72.6)	
**Liver metastasis**				
Absent	192	30 (15.6)	162 (84.4)	**0.005**
Present	13	6 (46.2)	7 (53.8)	
**Vascular invasion**				
Absent	178	26 (14.6)	152 (85.4)	**0.004**
Present	27	10 (37.0)	17 (63.0)	
**Tumor location**				
Head	139	23 (16.5)	116 (83.5)	0.580
Body/tail	66	13 (19.7)	53 (80.3)	
**Histological differentiation**				
Well	14	4 (28.6)	10 (71.4)	0.262
Moderate/poor	191	32 (16.8)	159 (83.2)	

Values in parentheses indicate percentage values. The bold number represents the P-values with significant differences.

**Table 2:** Univariate and multivariate analyses of prognostic parameters for survival in patients with pancreatic ductal adenocarcinoma (PDAC).

**Prognostic parameter**	**Univariate analysis**			**Multivariate analysis**		
	**HR**	**95% CI**	**P value**	**HR**	**95% CI**	**P value**
**Expression of LKB1** (low vs. high)	0.439	0.288-0.669	**0.000**	0.542	0.349-0.841	**0.006**
**Age** (≤65 vs. >65)	1.306	0.953-1.788	0.097	-	-	-
**Gender** (male vs. female)	0.908	0.660-1.249	0.553	-	-	-
**Clinical stage** (I vs. II vs. III vs. IV)	1.560	1.243-1.959	**0.000**	1.295	0.999-1.678	0.051
**Size** (≤2 cm vs. >2 cm)	1.850	1.219-2.806	**0.004**	1.628	1.063-4.494	**0.025**
**T classification** (T1 vs. T2 vs. T3 vs. T4)	1.449	1.143-1.836	**0.002**	1.163	0.896-1.511	0.257
**N classification** (absent vs. present)	1.592	1.132-2.239	**0.008**	1.327	0.928-1.898	0.122
**Liver metastasis** (absent vs. present)	3.700	1.953-7.007	**0.000**	2.318	1.157-4.646	**0.018**
**Vascular invasion** (absent vs. present)	1.491	0.951-2.338	0.082	-	-	-
**Tumor location** (head vs. body/tail)	1.042	0.743-1.459	0.813	-	-	-
**Histology** (well vs. moderate/poor)	1.503	0.766-2.950	0.236	-	-	-

HR: Hazard ratio; CI: Confidence interval. The bold number represents the *P*-values with significant differences.
