# Corrigendum: Decreased LKB1 predicts poor prognosis in Pancreatic Ductal Adenocarcinoma

**DOI:** 10.1038/srep15869

**Published:** 2015-10-29

**Authors:** Jian-Yu Yang, Shu-Heng Jiang, De-Jun Liu, Xiao-Mei Yang, Yan-Miao Huo, Jiao Li, Rong Hua, Zhi-Gang Zhang, Yong-Wei Sun

Scientific Reports 5: Article number: 10575; 10.1038/srep10575published online: 05272015; updated: 10292015

In the original version of this Article, there was a typographical error in Affiliation 1 which was incorrectly listed as ‘Department of Biliary-Pancreatic Surgery, Ren Ji Hospital, School of Medicine, Shanghai Jiao Tong University, 200240 Shanghai, P.R. China’. The correct affiliation is listed below:

Department of Biliary-Pancreatic Surgery, Ren Ji Hospital, School of Medicine, Shanghai Jiao Tong University, 200127 Shanghai, P.R. China

This error has now been corrected in the PDF and HTML versions of the Article.

